# The role of European municipalities and regions in financing energy upgrades in buildings

**DOI:** 10.1007/s10018-023-00363-3

**Published:** 2023-02-27

**Authors:** Marina Economidou, Nives Della Valle, Giulia Melica, Paolo Bertoldi

**Affiliations:** 1 European Commission, Joint Research Centre (JRC), Via Enrico Fermi 2749, 21027 Ispra, VA Italy; 2 European Bank for Reconstruction and Development, 5 Bank Street, London, E14 4BG UK

**Keywords:** Financing, Energy renovations, Buildings, Regions, Local authorities, Green Deal, G28, H31, Q48, R11

## Abstract

Energy efficiency in buildings has a central role to play in reaching the climate neutrality goal set by the European Green Deal. With detailed knowledge of their building stock and the profile of their occupants, regional and local authorities can forge an important link between financiers, industry professionals and homeowners to ensure the successful deployment of financial schemes that support the uptake of energy efficiency upgrades. This paper provides a first assessment of the role of regional and local authorities by reviewing relevant initiatives and programmes on energy efficiency. Based on a questionnaire completed for more than 150 schemes, it explores how European municipalities and regions stimulate energy upgrades in residential, commercial and public buildings through public financial support. It is found that 60% of the examined schemes are offered in the form of grants and subsidies, and 45% of them targeted residential upgrades. The use of EU cohesion policy funds in supporting regional schemes, and promotion of energy efficiency investments at local level through the European Covenant of Mayors initiative are also explored. In spite of possible resource limitations at this level of governance, regional and local authorities are in a good position to utilise European structural or research funds to develop financial schemes, as standalone programmes or blended with national ones, thus providing additional support and funds for deep renovations. The results suggest that energy efficiency in buildings has become an important part of local and regional strategies in several European countries, but could be further enhanced.

## Introduction

It is widely recognised that much of the energy used in buildings is largely wasted due to old construction practices, many of which preceded the adoption of energy performance standards (Economidou et al. 2020). With over 80% of today’s buildings expected to be in use in Europe by 2050, the building sector must be at the centre of decarbonisation efforts in the EU (EC 2016b, 2018). Energy renovations of buildings are singled out in the European Green Deal as a key initiative to drive energy efficiency improvements, boost economic growth, generate new jobs, support local businesses and strengthen industrial competitiveness (EC 2019b). These renovations or upgrades in buildings may comprise any intervention measures on the envelope of a building and/or its technical systems—often complemented with renewable energy technology installations and passive measures, which result into quantifiable energy savings. Energy upgrades through comprehensive renovations can also play a crucial role in the European recovery of the COVID-19 pandemic due to the labour-intensive nature of the building sector and large domination of local businesses (EC 2020c). Also, energy renovations lower energy bills and can reduce energy poverty (EC 2019b), which is recognised as a major issue in the European context, concerning individuals and households unable to adequately heat or cover the basic energy services in their homes (EC 2020b). Energy poverty faced by individuals and households that are not able to adequately heat or cover the basic energy services in their homes is recognised as a major issue in the European context.

The European Commission published its Renovation Wave Strategy in October 2020 with the aim to improve the energy performance of buildings across Europe (EC 2020a). To pursue the Green Deal ambition and to kick-start the post-COVID-19 recovery, the Commission has set out the goal of doubling the renovation rate in its dedicated recovery plan. The strategy is expected to rely on measures agreed under the Clean energy for all Europeans package, notably the requirement for each EU country to publish a long-term building renovation strategy, several aspects of the amending Directive on the Energy Performance of Buildings and building-related aspects of each Member State’s national energy and climate plans (Directive (EU) 2018/844 (2018); Regulation (EU) 2018/1999 (2018); Economidou et al. (2022)). This, in turn, requires the uptake of financing and the mobilisation of multiple actors at all governance levels, from municipalities to national and international jurisdictions.

There are several well-documented economic, financial, institutional, structural and behavioural barriers to energy efficiency in the building sector (Jakob 2007; Gillingham et al. 2009; Economidou et al. 2011; Wilson et al. 2015; Palm and Reindl 2018; Ebrahimigharehbaghi et al. 2019; Bertoldi et al. 2021; Della Valle and Bertoldi 2022). Renovation decisions entail complex and multi-faceted processes, subject to various influencing factors beyond technical issues, and policy interventions are often deemed necessary to support decision makers in various stages of the renovation journey. Financial schemes or instruments for energy renovations in buildings are widely recognised as a key policy instrument to address upfront cost barriers, thus supporting the push towards the transition in the sector (EC 2016a). They can take the form of non-repayable rewards, debt financing, equity financing, and they can range from well-established and traditional mechanisms such as subsidised loans to emerging and new models such as on-bill programmes (Bianco and Sonvilla 2021; Bertoldi et al. 2021).

So far, existing related literature has provided an overview of the potential of financial and fiscal mechanisms for energy efficiency improvements in buildings in Europe at the national level (Maio et al. 2012; Economidou and Bertoldi 2014; EEFIG 2015; Brown et al. 2019). In particular, many of the aforementioned studies investigate the ongoing national efforts in stimulating investments in building renovations.

However, many of the current policies and measures also take place at regional and local levels. In addition, regional and local authorities are in a position to forge a unique link with European citizens that makes their involvement critical in delivering climate and energy targets (Kern 2019). As an example, regional and local authorities can actively nurture behavioural change and ensure participation by citizens and local businesses in the energy transition (Della Valle et al. 2021). Notably, with detailed knowledge of their building stock and the profile of their occupants, regional and local authorities can also forge an important link between financiers, industry professionals and homeowners and ensure the successful deployment of financial schemes that can support the uptake of energy efficiency upgrades (Bertoldi et al. 2021).

Despite their key role in supporting the uptake of energy efficiency upgrades, so far there has been little focus in the literature on the local and regional efforts in stimulating investments in building renovations.

Our study aims to fill this gap in the literature, by providing a first assessment of the role of municipalities and regions in stimulating energy upgrades in residential, commercial and public buildings in Europe. Against this background, the paper addresses the following research questions:

- What current practices do European municipalities and regions deploy to support financing energy upgrades in buildings?
- What unique features do they bring, and are there any good practices?
- How effective are regional and local financial schemes?

To support this goal, a questionnaire was specifically designed and distributed to regional and local experts in all EU27 countries.^1^ The survey was then complemented by a review of national, regional and local reports including regional programmes or projects funded under Cohesion Policy funds, and European voluntary initiatives such as the Covenant of Mayors for Climate and Energy (CoM).

The European Commission actively promotes energy efficiency at regional level through its Cohesion Fund (CF) and European Regional Development Fund (ERDF). In particular, the CF supports Member States with Gross National Income per inhabitant less than 90% of the EU average, and aims to reduce economic and social disparities and promote sustainable development; the ERDF aims to strengthen economic and social cohesion in the European Union by correcting imbalances between its regions (Regulation (EU) No 1303/2013 (2013); Regulation (EU) 2021/1058 (2021)).

The CoM is one of the major European initiatives that acknowledges the crucial role of regions and local authorities and, for this, continues to receive strong political support. In a first phase, the CoM encompassed a minimum target of 20% GHG emission reduction by 2020, which was later on raised to 40% by 2030 and then to climate neutrality by 2050.^2^

In particular, the CoM, which has engaged with local governments in the climate challenge since 2008, has enabled several successful policy actions in promoting energy efficiency investments, including actions of financial and fiscal nature (Palermo et al. 2020). Within the CoM initiative, local authorities may also benefit from guidance and support from public authorities at a higher territorial level (such as regions and provinces) involved as Covenant Territorial Coordinators (CTCs) (Melica et al. 2018). Among other things, CTCs may provide support by identifying possible financing sources and mechanisms for the implementation of actions planned by local authorities, including on energy efficiency in public and private buildings. In addition, some CTCs also act as managing authorities, responsible for the implementation of operational programmes under Cohesion Policy. In this context, some CTCs have used Cohesion Policy funds to support the development of local Sustainable Energy and Climate Action Plans and/or to finance the implementation of SECAPs actions in various sectors, thus potentially increasing the uptake of ERDF and CF in their territories. In other cases, CTCs have been able to aggregate project initiatives from municipalities developing investment programmes of the required size to apply for support through the ELENA facility and loans from the European Investment Bank (EIB) (Lombardi et al. 2016; European investment Bank 2022).

By analysing the data collected from the survey, the CoM and the Cohesion Policy Funds, the paper provides a first qualitative assessment of the potential that regional and local financial and fiscal instruments play in stimulating energy upgrades in Europe. In particular, to assess financial schemes and identify good practices, we provide the current state of play in relation to local and regional energy renovation programmes in residential, commercial and public buildings in the EU, and use a set of criteria based on design, implementation and impact criteria, such as funding sustainability, scalability and success at addressing hard-to-reach groups (Economidou et al. 2019). Finally, we compare local and regional schemes with national ones to reveal comparable design and implementation features.

The research, therefore, extends its scope to regional and local efforts with the aim to provide a far-reaching overview of energy efficiency financing at all governance levels. Whilst the results presented here are non-exhaustive, this study represents the first of its kind in that it covers the regional and local governance levels which are still not fully examined in the literature.

The structure of the paper is as follows. Section 2 describes the methodology deployed and the questionnaire used to collect data. Section 3 provides a summary of the EU support through ERDF and Cohesion Funds in energy efficiency at regional level, and an overview of financial instruments adopted at local level within the CoM context. Section 4 summarises all programmes identified in our survey, including links with EU supported schemes, and Sect. 5 discusses the research questions based on the study findings. Policy conclusions are drawn in Sect. 6.

## Methodology and data sources

This research focuses on regional and local financial schemes on energy efficiency. These are defined as schemes administered by regional and local authorities, which may be funded from different streams of funding including local, regional, national and international sources, and disbursed to beneficiaries that fall within the jurisdiction of the authority in question.

A questionnaire survey was conducted in 2020 with the aim to collect information on financial schemes run by regional and local governments across the EU. The work builds from previous studies focussing at national schemes carried out by Economidou and Bertoldi (2014) and Economidou, Todeschi and Bertoldi (2019). These studies investigated general practices deployed by individual Member States in supporting energy renovations through public and private financial aid.

The questionnaire was designed to gather information on the main design features, implementation details and outcomes of financial schemes supporting energy upgrades in residential, commercial and public buildings. The information collected in the questionnaire is shown in Table 1. This included: 1. general information such as geographical scope and programme website, 2. design features such as policy type, eligibility conditions and minimum energy efficiency criteria, 3. various implementation details and 4. outcomes. The latter covered qualitative information on the level of achieved impact (low, medium, high) and quantitative data such as number of buildings benefitting from the scheme and achieved energy savings. By collecting both quantitative and qualitative information, the impact can be reviewed and verified across different schemes. The questionnaire was shared with a total of 267 experts in mid to end of 2020 and a total of 78 respondents participated in the survey. The majority of the experts were affiliated with regional energy agencies and local departments that handle energy renovations and building-related topics and the remaining ones one-stop shops (OSSs) and other types of specialized organisations.

**Table 1:** Information collected in questionnaire

General information	Name of scheme Geographical level [Regional, Municipality/city, Combination of above, Other] Name of region or city, website
Policy design features	Type of policy Targeted sectors Beneficiaries, eligibility conditions, dissemination, supported interventions, minimum energy efficiency criteria
Implementation details	Implementation period, implementation body, brief description, dissemination, budget, funding sources
Outcomes	Level of achieved impact; Total number of buildings, interventions, applications, etc.; Achieved energy savings, greenhouse gas (GHG) reduction and other benefits

To complement data from the survey, additional sources and initiatives were reviewed.

Additional data were retrieved regarding EU Cohesion Policy, since this has long supported regions and municipalities in the shift towards low-carbon economy, including through energy efficiency, through financial allocations. For this study, the Cohesion data catalogue (ESI Funds Open Data Platform—https://cohesiondata.ec.europa.eu) was used to extract data and explore how these funds have been used by regions for energy efficiency investments in residential, commercial and public buildings in 2014–2020. More particularly, only data under the theme “Low-Carbon Economy” (Theme 04) was selected, given that the European Structural and Investment Funds (ESIF) under this theme invest in a range of priorities to support the shift towards a low-carbon economy in all sectors, including energy efficiency.^3^ Then, from this selection, we further refined data extraction, by selecting data regarding ESIF funds specifically promoting interventions that support energy efficiency investments in:

i. Public buildings (Dimension 13),
ii. Residential buildings (Dimension 14),
iii. Small and medium enterprises (Dimension 68),
iv. And large enterprises (Dimension 70).^4^

Subsequently, we extracted data on the two main types of ESIF funds that directly support building renovations at regional level: the European Regional Development Fund (ERDF) and Cohesion Fund (CF), which provide grants or other financial instruments, wherein these latter can support national and/or regional programmes depending on the scope set by each Member State.^5^ Finally, as this aim of the study is on the local and regional level, we checked the coverage of all programmes resulted from the search above using the Atlas of Operational Programmes adopted by the European Commission and selected regional programmes only (https://ec.europa.eu/regional_policy/en/atlas/programmes).

Regarding the CoM initiative, we exploited the data falling under the strategies and measures to reduce energy consumption and carbon emissions (Kona et al. 2018). The data were analysed for the EU27 countries, with the aim to gain insights on the extent and the types of financial mechanisms adopted by signatory municipalities across the EU. In particular, the analysis used as main source of information the database of mitigation actions—focussing on energy efficiency and local renewable energy sources under the *Financing and provision* mode of governance for the *building sector*—reported by municipalities for the achievement of their 2030 GHG emission reduction target. A similar analysis was carried out on the database of mitigation actions reported for the achievement of the 2020 target, to derive a temporal comparison of the reported mitigation actions.

Finally, we analysed the schemes resulted from the survey identifying (i) how much and which types of ESIF have been exploited, (ii) whether the schemes targeting the municipality/city level fall under the CoM action plans, and (iii) good practices. Good practices were identified based on four criteria: (1) ambition of energy efficiency upgrades, (2) outreach to hard-to-reach groups, (3) funding continuity and sustainability and (4) innovative features. Whilst there is no widely accepted methodology in the literature on how to identify good practices or criteria for the evaluation of energy efficiency policies, there are several studies focussing on good policy practices (Boza-Kiss et al. 2013; de Melo et al. 2013; Tholen et al. 2013; Spyridaki et al. 2016; Broc et al. 2017; Economidou et al. 2019). To facilitate a meaningful discussion, the rationale behind the selection of the four criteria can be summarised as follows:

- Ambition of energy efficiency upgrades: As deep renovations delivering more than 60% energy savings occur in around 0.2% of the building stock per year, policy intervention to promote deeper renovations is crucial (EC 2019a). Moreover, the risk of “locking in” energy savings through suboptimal renovations is a major decarbonisation risk as suboptimal renovations defer the opportunity to exploit the full energy saving potential of a building to a possibly much later point in the future.
- Outreach to hard-to-reach groups: The just transition towards climate neutrality is a key political priority, highlighting the need to reach out to vulnerable groups that traditionally have no means or funds to engage in green projects (Bouzarovski et al. 2012; Kyprianou et al. 2019; Della Valle and Czako 2022). Vulnerable groups in this context include low-income households, social housing associations, condominium multi-owners, tenants, and SMEs.
- Funding continuity and sustainability: This includes continuity through long-lasting commitment by governments, use of sustainable funding mechanisms (e.g. revolving funds) and diversification of funding sources, e.g. by blending various sources at different levels of governance, or by earmarking funds from fossil fuel taxation to support decarbonisation efforts (Marron and Morris 2016).
- Innovative features: Such innovative features can offer scalability with the aim to address some of the most prominent barriers to energy efficiency.

## EU support and initiatives

### Regional support through ERDF and cohesion funds

In the period 2014–2020, nearly EUR 25 billion have been allocated on energy efficiency investments in buildings, with 69% stemming from EU Cohesion Policy funds.^6^ Nearly half of the total funds (EUR 12.7 billion) were allocated to investments in public buildings.

**Fig. 1:**
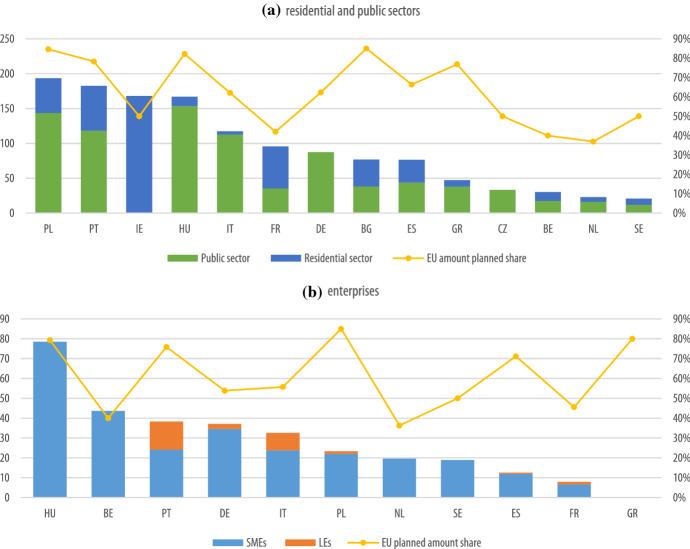
Total amount (in EUR) per capita including national co-financing, (in EUR) per capita spent on energy efficiency investments in: (**a)** residential and public sectors and (**b)** enterprises based on latest ESIF database – latest update June 2021 (EU shares represent the amount of EU amount planned versus the total amount planned incl. co-financing in the programmes in each country)

In terms of the share of regional and national programmes supported by ERDF and CF under the category “Low-Carbon Economy” for energy efficiency in residential and public buildings, Estonia, Croatia, Lithuania, Luxembourg, Latvia, Malta, Romania, Slovenia and Slovakia earmarked their cohesion policy funds in national programmes only. Other countries, such as Czech Republic, Spain, Greece, Hungary, Poland, and Portugal allocated the cohesion policy funds for national programmes only for a small share. With the exception of Ireland, which exclusively used ERDF funds for regional programmes in the residential sector, generally all countries used ERDF funds for regional programmes predominantly in the public sector. A closer look at regional programmes on residential and public buildings (Fig. 1a), suggests that Poland followed by Hungary, Portugal, Italy and Germany stand out as the countries with the highest planned amounts per capita for energy efficiency investments in the public sector. Conversely, Ireland, Portugal, France and Poland, followed by Belgium and Spain stand out as the countries with the highest planned amounts per capita for energy efficiency investments in residential buildings. In all cases, the EU planned amounts per capita contributed proportionally to the planned amounts by each country, but this ranged from 37% in case of the Netherlands to 85% in Poland and Bulgaria.

In terms of the share of regional and national programmes supported by ERDF and CF under the category “Low-Carbon Economy” for energy efficiency in enterprises, Austria, Denmark, Croatia, Lithuania, Latvia, Malta and Slovakia invested their allocated Cohesion Policy funds only in national programmes, Italy and Portugal in both national and regional programmes, and all other remaining countries in regional programmes only. The majority of countries used ERDF funds for regional programmes for small and medium enterprises (SMEs), with the exception of Germany, Spain, France, Italy, Poland and Portugal, which also invested ERDF funds for large enterprises (LEs). Within these programmes (Fig. 1b), Hungary followed by Belgium, Germany, Portugal and Poland stand out as the countries with the highest planned amounts per capita for energy efficiency investments in SMEs. Conversely, Portugal and Italy stand out as the countries with the highest planned amounts per capita for energy efficiency investments in large enterprises. In all cases, the EU planned amounts per capita contributes proportionally to the planned amounts by each country, but this can range from 36% in case of the Netherlands to 85% in Poland. This variation is linked to different co-financing rates according to the category of regions.

### Covenant of mayors initiative

**Fig. 2:**
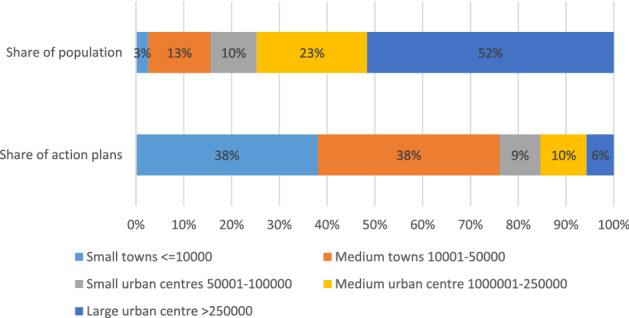
Share of action plans and population as a function of the size of the local authority under the CoM initiative

The analysis of 176 action plans with a 2030 target submitted until August 2019 by CoM signatories in the EU-27, covering about 11 million inhabitants, included 1060 financial instruments targeting the building sector. These cover instruments deployed at various governance levels including energy supplier schemes which are implemented at national level (Fawcett et al. 2019). Figure 2 shows the analysed action plans and the population they cover, as a function of the size of the local authorities. Although 76% of the action plans come from small and medium towns, they only account for 16% of the population, while 6% of the action plans come from large urban centres and cover more than half of the population.

**Fig. 3:**
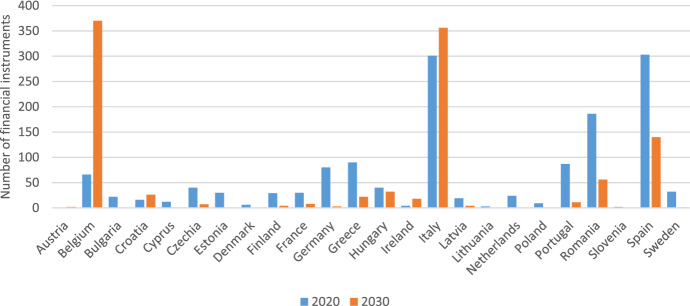
Overview of CoM financial instruments by reporting period and country

The financial instruments adopted and reported by local authorities for the achievement of the 2020 and 2030 targets within the CoM are depicted in Fig. 3. Overall, the number of financial instruments reported to achieve the 2030 target seems to follow the trend of the instruments reported to achieve the 2020 target, with the exception of Belgium and Italy —which increased their commitment to finance energy efficiency, and of Greece, Portugal, Romania and Spain —which instead reported fewer financial instruments.

**Fig. 4:**
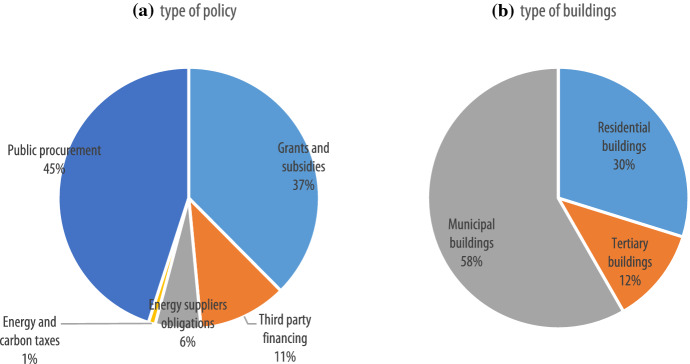
Overview of CoM financial instruments by (**a)** type of policy and (**b)** type of building (2030)

Diving into the financial instruments to achieve the 2030 target across the EU (Fig. 4), it can be seen that public procurement represents 45% of the total number of adopted financial instruments, followed by grants and subsidies (37%), third-party financing (11%) and energy suppliers’ obligations (6%). Energy and carbon taxes represent only 1%. The reported financial instruments are mostly concentrated on municipal buildings (58%), followed by residential buildings (30%) and tertiary buildings (12%). Across countries, there is no variation with regard to the priority given to public procurement, grants and subsidies and third-party financing (see Annex Table 4 for breakdown by country). The only exceptions are represented by Austria and Romania, in which grants and subsidies is the most preferred instrument, and, in which third-party financing is preferred to public procurement and grants and subsidies, and Spain and Croatia, which also opt for the energy and carbon taxes. In terms of building types, there is no variation across countries, with the exception of Romania, in which tertiary buildings is the most targeted sector, followed by municipal buildings and residential buildings, Austria, which exclusively focuses on the residential sector, and Latvia and Sweden, which exclusively focus on public buildings.

**Fig. 5:**
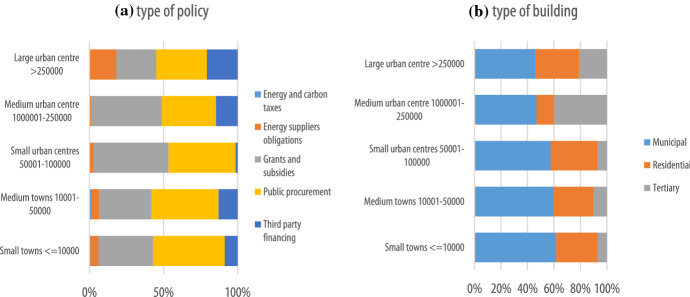
Type of policy and type of building type of CoM financial instruments according to size of local authority (2030)

Looking at the types of policy by size of local authority (Fig. 5), it appears that for small and medium towns (up to 50,000 inhabitants) public procurement is the preferred instrument, while for small and medium urban centres (between 50,000 and 250,000 inhabitants) a slight preference for grants and subsidies is observed. The share of energy suppliers’ obligations and third-party financing among the financing instruments increases for large urban centres. Finally, there is no variation across local authorities of various sizes in terms of the most targeted sector: municipal buildings appear to be always the preferred sector targeted by financial instruments, although the share of financial instruments targeting private buildings seem to increase in urban centres above 100,000 inhabitants.

## Overview of European local and regional schemes in 2020

**Fig. 6:**
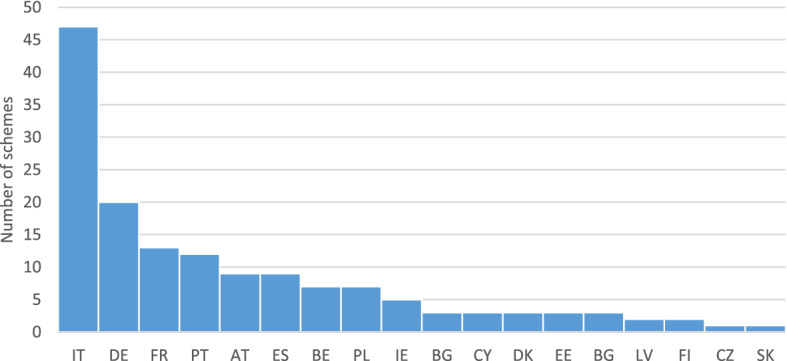
Number of regional and local schemes collected in the survey by country

The survey covered 151 local and regional schemes across the EU27, with the largest number of schemes located in Italy, Germany, France, Spain and Portugal (Fig. 6). The promotion of energy upgrades in buildings depends on the governance structure in each EU country, whereby some countries may choose to rely exclusively on the use of financial instruments at national level, while others offer multi-level support with a combination of schemes at national, regional and local levels. Based on the survey findings, nine countries fell in the first group of countries. Of the 27 countries examined, it was found that Croatia, Greece, Hungary, Luxembourg, Lithuania, Malta, Romania, Slovenia and Sweden did not actively rely on any regional or local schemes. In some cases, national schemes may have a regional dimension, e.g. with funds managed at national level, but disbursed at regional level (e.g. Greece and Croatia). Even though a few local schemes at municipality level have been identified in Bulgaria, Croatia, Czech Republic, Cyprus, Latvia, Lithuania and Slovakia, this group of countries largely rely on national schemes. On the other end of the spectrum, Austria, Belgium, Germany and Italy have a strong regional governance structure, with many regional schemes in place. In total, 109 programmes (representing 64% of total) were at regional level, 41 (24%) local level, 19 (11%) at national level and 1 at a hybrid level. The latter concerns a Spanish scheme managed by the regional administration, but directed to municipalities.

### Types of regional and local schemes

**Fig. 7:**
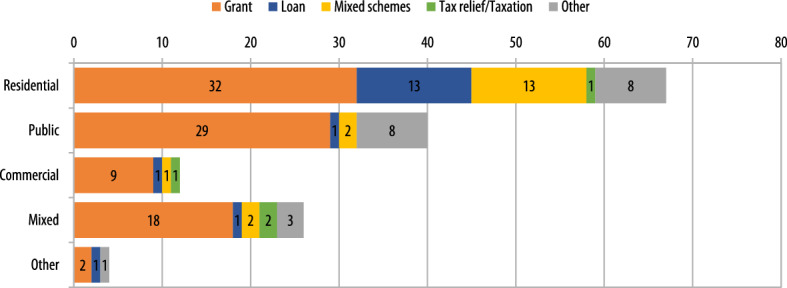
Regional and local financial instruments collected in this study by type of policy and building type

A summary of the regional and local schemes collected in the survey by country are shown in Fig. 7. Public support at local and regional levels is predominantly offered in the form of grants and subsidies. This is also a trend observed at national level (Economidou and Bertoldi 2014; Economidou et al. 2019; Bertoldi et al. 2021). In summary, a total of 90 schemes (or 60% of the total) were offered in the form of grants/subsidies, followed by loans (17 schemes or 11% of total), mixed schemes (18 schemes or 12% of total) and tax incentives (4 schemes or 3% of total). In most cases, mixed schemes combined loans with grants and/or subsidies. Other schemes included reward programmes, technical assistance instruments, general support, and the deployment of specific strategies. Grants and subsidies was a type of instrument deployed in Austrian, Belgian, Bulgarian, Cypriot, German, Spanish, French, Croatian, Irish, Italian, Latvian, Polish, Portuguese and Slovakian regions and/or municipalities. Loans and soft loans were available in Belgian, Cypriot, Danish, Spanish, French, Italian, Dutch, and Polish regions. Mixed schemes were identified in regions in Austria, Belgium, Germany and Italy, while tax incentives were rarer (only found in the region of Puglia in Italy and Riga in Latvia). Just less than a half of all schemes (68 schemes or 45% of total) were designed to support energy upgrades in the residential sector alone and just over a quarter (40 schemes or 26%) in the public sector (Fig. 7). Some residential schemes targeted energy efficiency improvements in single family houses only, others focussed exclusively on multi-family houses and others covered all residential types. Commercial buildings, which were exclusively addressed by 12 schemes (or 8% of the total), included small and medium enterprises, the hospitality sector and other businesses. Mixed schemes, representing 17% of the total, combined residential with commercial buildings, residential with public buildings or other combinations.

### Links with EU supported schemes

Of the 151 identified schemes through the survey, 51% were declared to be supported through EU funds (15% did not know, while 34% were funded through other sources). Italy had the highest percentage of EU supported schemes, followed by Germany, France, Portugal and Spain.

**Fig. 8:**
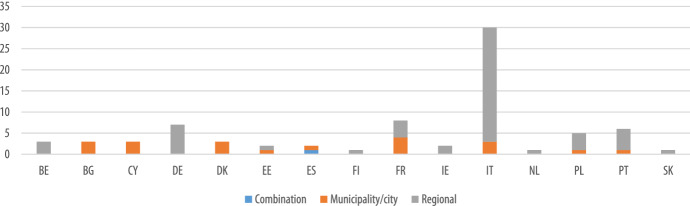
Overview of geographical level of EU supported schemes

The survey was mainly addressed to regional authorities across Europe, resulting in a higher percentage of regional schemes. The survey results confirm this, as it was found that EU funds were mainly exploited for regional schemes across all Member States, with the exception of France, Denmark, Cyprus and Bulgaria, for which the EU funds were exploited mostly for municipal schemes (Fig. 8).

**Fig. 9:**
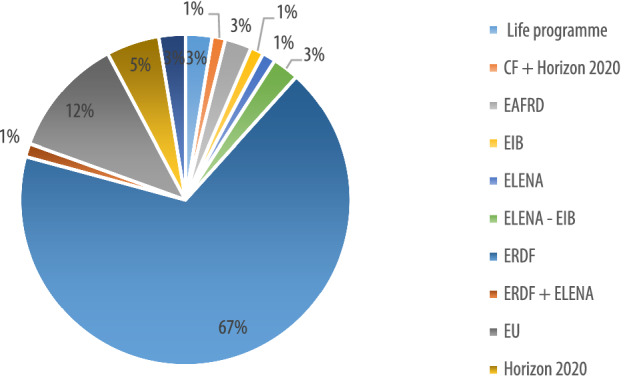
Overview of types of EU funds

Across the EU funds that were explicitly named (12% were generally referred as EU funded), ERDF represents the most exploited option (67%), followed by Horizon 2020 (5%) and Elena (3%) (Fig. 9).

Similarly to the general findings on EU schemes, when looking at schemes funded only through ESIF, the majority (85%) were used for regional schemes, and only a few for municipal schemes (13%) or a combination of both (2%).

**Fig. 10:**
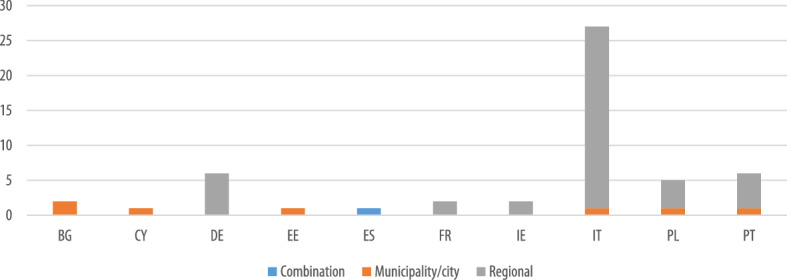
Overview of geographical level of ESIF-funded schemes by MS

Overall, ESIF funds were mainly exploited for regional schemes across all Member States, with the exception of Bulgaria, Cyprus, and Estonia, for which ESIF funds were earmarked in municipal schemes, and Spain, for which ESIF funds were exploited for schemes dedicated to both regions and municipalities (Fig. 10). However, as in our survey regional authorities represented a higher share of respondents than municipalities, we cannot rule out a bias in these results due to oversampling. For this reason, a closer look to the CoM database enabled us to enrich the findings from a municipal perspective.

#### Financial schemes at city level and links with CoM action plans

**Fig. 11:**
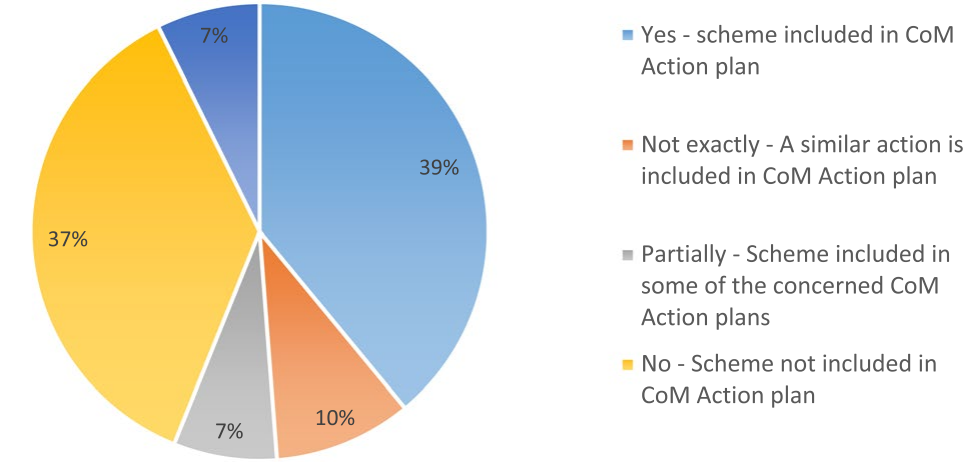
Financing schemes at city level included in CoM Action plans

Looking specifically at the 41 financing schemes implemented at the municipality/city level, we note that more than half of them showed a clear link with the Action plan developed by the concerned cities in the context of the Covenant of Mayors (Fig. 11):

- 16 schemes are presented in the action plan and described with sufficient level of details. For example the Écoréno'v scheme by Grand Lyon is a very prominent action of the plan, and is accompanied by other supporting actions regarding communication and awareness raising; similarly, the action plan of Berlin (Berlin Energy and Climate Protection Programme 2030) makes a clear reference to the four schemes identified through the survey and also highlights them as best practices, combining them together with awareness raising and regulatory measures, in the broader context of the city aiming to cut carbon emissions by 95% by 2050.
- 4 schemes are mentioned in general terms in the action plan: for example the Plan of Brest Metropole describes a portfolio of actions aimed at fostering energy renovation of buildings in the lowest energy classes, but does not mention explicitly the scheme identified through the survey.
- 3 schemes (decided at regional or national level) are targeting a defined subset of cities, but are included in the action plans of just a few of them and/or are mentioned in very general terms as a possible source of funding for energy renovation interventions: this is the case of ERDF grants in Bulgaria and Sicily.

About 37% of the schemes (15) were not found in the CoM Action plan of the concerned cities. This could be due to a temporal mismatch between the development of the action plan and the deployment of the scheme: for example the city of Riga approved its CoM Action plan in 2014, while the grant scheme identified through the survey is from 2020.

Also, action plans often present the strategic vision of local authorities towards decarbonisation and climate neutrality, and describe the main actions to be implemented with different levels of details from city to city: in some cases, cities describe in general terms the actions they plan in various sectors, and leave the identification of financing sources and schemes for a later stage.

Only 3 schemes out of 41 referred to cities with no action plan submitted to the Covenant of Mayors.

### Policy design, implementation and outcome features

Table 2 provides an overview of the main design, implementation and outcome features of the collected regional and local schemes. In terms of policy design, minimum energy efficiency criteria were applied to just over two thirds of the schemes. These criteria were often expressed as a minimum share of energy savings, maximum energy demand expressed in kWh/m2, certain level of energy class improvement, compliance with national building codes for renovations or prescriptive-based criteria (e.g. heat pump efficiency factor or U-values for insulation). Upgrades of technical systems followed by insulation of building envelope and installations of RES systems constituted the most common interventions targeted by the schemes covered in our survey. Often, the schemes offered support for a combination of different interventions with over 80% of the schemes promoting multiple interventions per building. Beyond direct renovation support, financial support was also foreseen for services such as technical or legal assistance, energy audits, general repairs/renovation and consultancy fees. The target renovation level per building in terms of achieved energy savings, based on the expert judgement of the survey respondents, was declared as high (25% of all schemes), medium (31%), low (6%), while for 38% it was not possible to make any conclusions. Beneficiaries ranged from homeowners to private or social housing associations, and from landlords and tenants to public authorities and SMEs. Public buildings included city halls, educational-purposed buildings, social housing, offices and other buildings of non-residential use.

**Table 2:** Distribution of regional and local schemes by main design, implementation and outcome features

Policy design	Yes	No		Policy implementation	Yes	No
Application of energy efficiency criteria	103	48		Known budgetary sources	105	46
*of which:*				Secured EU support	77	74
Minimum energy savings	35			Disclosed implementation period	145	6
Energy class improvement	19			started before 2000	1	
National building codes	10			…between 2000–2010	8	
Maximum energy demand	4			…between 2010–2015	35	
Positive energy building	1			…between 2015–2020	78	
Prescriptive criteria	14			started after 2020	2	
Other	14			Known dissemination channels	143	8
Known intervention measures	146	5		*of which:*		
*of which:*				Website	103	
Upgrade of technical systems	90			Press release	52	
Insulation of building envelope	86			Mass campaign	28	
Installation of RES systems	61			Tailored advertising (by post, phone, etc.)	14	
Installation of control systems	46			Training or toolkit	6	
Others	35			**Policy outcomes**	**Yes**	**No**
Declared target renovation depth	92	59		Known qualitative impact	66	85
*of which:*				*of which:*		
high	38			High	32	
medium	47			Medium	22	
low	7			Low	12	
Disclosed beneficiaries	145	6		Known quantitative impact	91	60
*of which:*				*of which:*		
Homeowners	57			Number of buildings	77	
Landlords or tenants	21			Number of interventions	65	
Housing or social housing associations	22			Number of applications	65	
SMEs	11			Energy savings	66	
Business owners	14			GHG reduction	62	
Public authorities	63			Other benefits	51	

On implementation, responsible bodies in charge of disbursing funds and reaching out to target groups included local and regional authorities such as local government bodies, municipalities, regional directorates and public housing authorities. The survey respondents disclosed budgetary resources for about 74% of all regional and local schemes. Total budgetary resources ranged from several thousand euros in certain cases to hundreds of millions euros in others and just over half of all regional and local schemes received some form of EU support. In total, 8 schemes started sometime between 2000 and 2010, 35 between 2010 and 2015, and 78 between 2015 and 2020. A wide range of channels were chosen to disseminate information among interested beneficiaries, with websites being the most common one, followed by press release, mass campaign, tailored advertising, etc.

As shown in Table 2, our analysis has shown that the impact of 85 schemes or 56% of all regional and local schemes could not be described in qualitative terms (low, medium or high) by respondents due to lack of appropriate monitoring and assessment mechanisms. Of the remaining 44%, nearly half was deemed to be of high impact (32 schemes or 21% of total), a third of medium impact (22 schemes or 15% of total) and the rest 12 schemes of low impact. High impact measures typically corresponded to measures associated with annual energy savings of the order of 100 GWh, medium impact measures generating savings of around 50 GWh and low impact measures savings of 20 GWh or lower. The quantitative impact in terms of achieved energy savings was reported for 66 schemes, GHG reduction for 62 schemes and other benefits for 51 schemes. Other reported benefits covered renewable energy production in MWh, number of new jobs, economic savings, reduction of social costs caused by climate change, reduction in PM10 pollutants or NOx gases and renovated floor area. In several cases, other benefits were described in qualitative terms, e.g. improved thermal comfort, improved indoor climate, better accessibility to buildings, alleviation of energy poverty and positive impact on local businesses. The total number of buildings benefiting from the schemes as well as total number of interventions or applications have also been reported in many cases.

## Discussion

The discussion below is framed against the three research questions set in the Introduction (Sect. 1). Based on the analysis of all schemes, we summarise current practices that European municipalities and regions deploy to support financing energy upgrades in buildings, discuss unique features and good practices and draw key findings in determining the effectiveness of these schemes. Links between the analysis of ESIF, CoM, and survey results are also provided.

### Overview of current practices

Grants, the most popular type of financial support offered by regional and local authorities, typically subsidised renovation-related costs, or in limited number of cases energy audit costs and other consultancy support services. For renovation cost subsidies, grant intensity depended on the type of intervention or, more specifically, on the energy efficiency level to be achieved. Such examples included the Écoréno'v scheme in Lyon with grants of EUR 2000 per unit for interventions delivering 35% energy savings and EUR 3500 for interventions bringing the energy consumption to the level of “bâtiment basse consummation” (BBC) and additional support for the use of bio-based materials. The grant intensity of the BE2 scheme in the city of Milan varied significantly depending on the type of intervention, from 10% of the eligible costs in case of diesel boiler replacement with natural gas boiler to 70% when a fossil fuel boiler was substituted by heat pumps, micro-CHP, or solar thermal.

The analysis also showed that grants were generally associated with stringent eligibility conditions, for example linked to the type and/or age of the building or to the income of owners or occupants. For example, the Mur|Mur scheme from Grenoble Alpes Metropole during its first phase of implementation targeted condominiums built between 1945 and 1975 and during a second phase extended its scope to condominiums built before 1945 and after 1975. The Wärmeschutz im Gebäudebestand scheme from Hamburg targeted buildings with a permit approved before 1995. Another example was the Warmth and Wellbeing Pilot Scheme from Dublin, targeting vulnerable (0–12, or 55 +) persons who are in receipt of social support.

The analysis of loan schemes showed examples of how local and regional authorities collaborated with local or regional banks to offer attractive financial products to homeowners or SMEs. This included the Investment Bank Berlin Brandenburg’s Energetic Building Renovation offering low-interest loans by the KfW Bankengruppe (KfW banking group) with a further nominal interest subsidy. Another example was the “Gran Prestito” developed by the city of Parma in Italy together with a local bank, to offer a loan (no lien) of up to EUR 50,000 and tenor of up to 10 years for energy renovations aligned with national requirements and in support with the Italian tax deduction scheme (Bonifaci and Copiello 2017). The municipality of Frederikshavn in Denmark trained local banks to assist homeowners in securing financing following free energy advice and energy saving proposals provided by the local utility. With the financing in place, an OSS would then supervise the implementation of proposals and provide quality assurance of completed work (Bertoldi et al. 2021), showcasing how financing can be practically combined with measures aimed at raising awareness, building capacity and providing technical assistance. The Oktave scheme in Grand Est Region (France) provided OSS services covering advice, support and funding through third-party facility with social cooperative companies and banks (Mcginley et al. 2020). Finally, the Assen Service Costs model developed by the Dutch city of Assen offered an interesting case study in terms of support of home renovations towards zero-on-the-metre based on ‘object based-funding’ instead of personal-based funding, thus enabling renovations of apartment blocks as a whole, instead of single units.

Loan schemes blended with other products (mixed schemes) included the combination of revolving funds with grants and loans (e.g. Riga) and the Estonian Kredex grant scheme (which supports renovation to Class C) with a city subsidy aimed to reach Class A (Kuusk and Kalamees 2016; Hamburg and Kalamees 2019). The Riga revolving fund provided low interest and long-term loans to improve building envelope insulation and upgrade building technical systems of 6000 buildings built before 1996, in addition to grants to support project preparation, investment costs (rewarding the most ambitious projects) and low-income households. Beyond these, some schemes were classified under “other types” such as the "Aradippou Oxygen Rewards Card" Mechanism in Cyprus, which seeks to reward Aradippou residents who invest in household energy efficiency measures and solar photovoltaic (PV) installations. Under this mechanism, citizens are rewarded for their solar PV investments by receiving payments on a municipality-issued credit card called the “Oxygen Rewards Card”. The concept connects PV investments and associated emissions reductions by local citizens with actors purchasing carbon offsets on compliance or voluntary carbon markets.

Finally, a few examples of tax relief schemes were also identified. In Riga, apartment blocks, all facades of which have been insulated after its commissioning, or free-standing groups of premises (apartments) located in such a building are entitled to a 90% discount on the real estate tax. Another example is the Housing Plan in the Region of Puglia in Italy, offering tax relief for construction activities and architectural, energy and environmental quality improvements of existing buildings.

#### ESIF-funded programmes

While local authorities can play a key role in promoting energy efficiency as they are embedded in the local context and can identify local needs, they often lack the investment capacity to implement energy efficiency programmes. This can be overcome by providing access to international funds such as ESIF. However, often, local authorities are neither aware of the existence of these funds nor of how to use them for the financing of sustainable energy projects (Streimikiene et al. 2007).

We found that while many countries indeed have exploited EU schemes, notably the available Cohesion and ERDF funds, to fund regional programmes, other countries did not take fully advantage of them. In particular, the countries that exploited ESIF the most, with the exception of Poland and Slovakia, are also among those that promoted the largest number of schemes (Italy, Germany, Portugal and France). This suggests that for regional and local authorities to play a more effective role in promoting energy efficiency with their citizens, these funds could be used better and more active participation in relevant European-wide or other international initiatives should be activated.

Therefore, to make these available financing options more effective and actually used, tailored communication campaigns on these funds as well as dedicated trainings empowering regional and local authorities’ project competencies should be promoted.

#### Financial schemes at city level and CoM action plans

The results of the analysis in the CoM context shows that local authorities tend to put greater emphasis on municipal buildings (representing 58% of the planned financial instruments) and less on private buildings (with residential representing 30% and tertiary 12% of the analysed instruments).

A closer look at the CoM Action Plans of the cities targeted by the 41 schemes at municipal level shows that about 39% of the schemes were clearly mentioned in the action plans. This concerns notably bigger cities such as Berlin, Grand Lyon, Tartu and Vilanova de Gaia, which have longstanding experience in climate action and are more active in the Covenant of Mayors initiative. 54% of the schemes are either not mentioned at all in the action plan (15 schemes), or just referred to in general terms (7 schemes); although in some cases this might be due to a temporal mismatch between the action plan approval and the deployment of the scheme, it may also suggest that cities (especially those at the beginning of their climate journey) might not be fully aware of the available financing schemes for energy renovation of private buildings.

The Covenant of Mayors, being also a platform for the dissemination of best practices, has the potential to tap into the potential of local authorities to trigger energy renovation of private buildings and possibly increase the uptake of Cohesion Policy funds for energy efficiency.

### Identification of good practices

Examples of good practices identified in this analysis are outlined in Table 3. Firstly, the renovation ambition was found to be an important consideration in the studied financial schemes. Some schemes that stand out include the exemplary building programme in Centre-Val de Loire region in France, which provided grants to buildings that reach positive energy levels and the modernisation scheme in Hamburg which granted financial support for major energy upgrades of rental apartments under the condition of maximum annual energy demand of 90 kWh/m^2^.

**Table 3:** Examples of good practices identified in this analysis

Name	Geographical scope	Covenant involvement as signatory or CTC	ESIF support	Justification
***Criterion 1: Ambition level of renovation***				
Modernization of rental apartments	City of Hamburg (Germany)	Signatory	-	Final energy demand below 90 kWh/m^2^ per year
Exemplary buildings in energy efficiency	Region of Centre-Val de Loire (France)	CTC	Yes (ERDF)	Support to buildings that reach positive energy level
POR FESR 2014–20	Region of Piedmont (Italy)	CTC	Yes (ERDF)	Reduction of minimum 40% in energy consumption
H2020 SmartEnCity	Tartu (Estonia)	Signatory	-	Energy class A renovation
***Criterion 2: Hard to reach groups***				
Better Energy Warmer Homes	Border, Midland & Western Region (Ireland)	No	Yes (ERDF)	Grants to owners of privately owned homes built pre 2006 that are under low-income thresholds (e.g. in receipt of fuel allowance as part of the national fuel scheme, job seekers allowance for more than six months with a child under seven, working family payment, one-parent family payment, domiciliary care allowance or carer allowance)
Energy renovation of public housing	Brest métropole (France)	Signatory	-	Energy and technical renovation of units in collective and individual public housing (priority for energy label E, F and G)
Grants to promote operational resource and energy management optimisation	State of Lower Saxony (Germany)	No	Yes (ERDF)	Grants covering up to 70% of eligible costs in SMEs for the uptake of operational resource efficiency and energy management measures
Provincial incentives for the energy rehabilitation of condominiums	Alto Adige Region (Italy)	No	-	Incentives of 70% for interventions in condominiums with at least 5 units exclusively owned by 5 persons or not-for profit entities
***Criterion 3: Funding continuity and sustainability***				
Midlands Retrofit Scheme	Midlands IE063 (Ireland)	No	-	Funding secured through allocation of EUR 20 million Carbon Tax revenues
Territorial subsidies coupled with national white certificate scheme to support Ardèche in their energy transition	Ardèche (France)	No	-	Significant territorial grants to their communities through integration with national white certificate scheme
Limburg sustainable home investment scheme	Province of Limburg (Netherlands)	No	-	Combination of multiple streams of funding (e.g. European Investment bank and provincial resources) using a revolving funding mechanism
***Criterion 4: Innovative features***				
LEMON programme	Emilia-Romagna (Italy)	CTC	-	Combination of grant, loan, energy performance contracts for energy efficiency improvements in social housing, integrating multiple sources of funding
Oktave	Region of Grand Est (France)	No	-	Innovative financial model for renovation towards zero-on-the-metre without use of personal loans, funded through homeowner associations (HOA) and supported by guarantee fund by regional authorities. Bank loan without collateral. Home owners pay a regular fee to HOA
Oxygen Rewards Card mechanism	Aradippou (Cyprus)	Signatory	-	Reward of citizens for EE and solar PV investments through payments on municipality-issued credit card
BE REEL! C13	City of Louvière (Belgium)	Signatory	-	Set up of innovative financing scheme via third-party investment that combines 0% loan from the Walloon region

Interesting design features was the use of well-defined eligibility criteria (e.g. beneficiary groups in the Irish Better Energy Warmer Homes or types of condominiums, which would normally not be outside the scope bankable products in the Italian region of Alto Adige). In the commercial sector, relevant schemes included high intensity grants for SMEs (e.g. in the German State of Lower Saxony).

In relation to the third criterion, it was found that carbon tax revenues were directly used to fund the Midlands Retrofit Scheme in Ireland. Through the allocated EUR 20 million carbon tax revenues, the scheme focussed in areas with high fossil fuel use and negative community impact due to the closure of peat-fired power stations. The combination of national and regional efforts to optimise existing schemes was adopted by the department of Ardèche (France) through the integration with the national white certificate scheme, giving the opportunity for an early stage involvement and providing valuable inputs to improve the quality (Osso et al. 2019). Other interesting cases included the revolving fund in Limburg combining multiple streams of funding from the European Investment bank and own provincial resources and the Investment Bank Berlin Brandenburg enabling the combination of three funding programmes.

Finally, technical assistance, third-party services, ESCO participation and one-stop-shop services were some of the main innovative features. This include Oktave’s full OSS services and bridge funding through a dedicated fund to tackle upfront costs of renovations and the Less Energy More Opportunities programme (LEMON) in the Italian Region of Emilia-Romagna combining grant, loan, technical assistance and energy performance contracting in social housing. HolaDomus implemented by EuroPACE —a European project designed to make home renovation simple, affordable and reliable (Bertoldi et al. 2021)— offers OSS services with the participation of Municipality of Olot and GNE Finance.

### Qualitative evaluation of effectiveness and outcomes

Annex Table 5 provides an overview of schemes that were reported to be of “high” impact by respondents and at the same time were associated with large amount of achieved energy and/or GHG savings as well as significant budgetary resources. As expected, large-scale programmes in major cities and regions are associated with a high impact in absolute terms, and their impact is also confirmed by the answers to the qualitative impact question included in the survey. Despite this, cross-comparisons and assessment of effectiveness across all schemes including smaller scale ones, or indeed definition of relevant benchmarks are rarely possible at the regional/local level due to a number of reasons. Firstly, our survey results confirm various gaps identified by previous studies on the quality and availability of quantitative data on outcomes, in particular with regards to effectiveness or cost-effectiveness of energy efficiency programmes (Wade and Eyre 2015; Broc et al. 2017). This may often stem from lack of resources to carry out in-depth ex-ante and ex-post impact evaluation studies. Collecting and aggregating results across different programmes is exacerbated by the absence of standardised monitoring, reporting and verification methods. These issues, coupled with the uncertainty around underlying definitions, assumptions and baselines, pose major obstacles in undertaking quantitative assessments and engaging in meaningful comparisons across different schemes. Cost-effectiveness is also complex to assess due to important data gaps, lack of methodological clarity and non-harmonised approaches in the way that energy savings and cost-related data are computed. This confirms that, rather through the comparison of physical outcomes, the discussion of good practices can currently be guided mainly by the identification of exemplary design and implementation features.

## Conclusions

The local and regional financing schemes analysed in this study confirm the need to consider different governance levels when investigating the promotion of energy efficiency upgrades in the EU. By exploiting data from the questionnaire distributed to regional and local authorities and experts, and from EU cohesion policy funds and CoM initiative, we found that that the promotion of energy efficiency improvements in buildings is carried out at different governance levels, depending on the governance structure of the country in question. Some EU Member States with strong national governance relied exclusively on the use of financial instruments at national level, while others such as Austria, Belgium, Italy and Germany offered multi-governance support at national, regional and local levels. Several local financial schemes were combined with regional and/or national ones, enabling the delivery of higher savings and encouraging deeper renovations in certain cases. In addition, regional and local authorities often offered advisory services to citizens aiming at accelerating the uptake of national financial instruments and prioritising interventions targeting the most inefficient buildings. The analysis of the collected schemes also illustrated cases in which local authorities worked with banks to develop suitable products, supported the setup of OSSs, built capacity of professionals in the construction sector and provided energy advice to homeowners.

National Recovery and Resilience Plans (NRRPs) could have also been a valuable source of data regarding investments for the acceleration of building renovation. However, this research was conducted before the NRRPs become available. Therefore, an avenue for future research would be to investigate also the amount of resources allocated by Member States through the Recovery and Resilience Facility to the renovation of buildings. Whilst this analysis is non-exhaustive, a comparison between exemplary local/regional schemes with national schemes revealed comparable design and implementation features (Economidou, Todeschi and Bertoldi 2019; Bertoldi et al. 2021). This shows that regional and local schemes can retain the same or in certain cases higher ambition levels, while forging a close link with beneficiaries, especially vulnerable groups. Several of these schemes supported ambitious energy efficiency upgrades by setting strict energy performance requirements and supporting energy upgrades in buildings occupied by vulnerable groups, low-income households, condominium multi-owners, tenants, and SMEs. Others offered long-term continuity based on lasting public commitment or more sustainable funding structures e.g. through the use of revolving funds, diversified funding sources and earmarked funds from taxation of fossil fuel use. Technical assistance, third-party services and involvement of ESCOs were features in some cases (Bertoldi and Boza-Kiss 2017). Tax relief schemes at regional level, however, were rare as they typically fall under the competence of national authorities.

Our analysis has also identified schemes generating significant energy savings, reduction of GHG emissions as well as investments. The impact and cost-effectiveness of financial schemes could be studied in more detail in the future under the condition that more comparable data are made available through the implementation of harmonised definitions and methodological approaches. Nevertheless, limited resources at local and regional levels may have a detrimental effect on the overall impact. Due to lower budgetary support, regions and municipalities may not have the same outreach and, thus, impact in absolute terms as national schemes do. This highlights even further the need of available EU resources for regions and local authorities for the uptake of energy efficiency investments.

Better utilisation of available national, EU and international (e.g. EIB, World Bank or European Bank for Reconstruction and Development) funds, and more active participation in relevant European-wide or other international initiatives is thus recommended in the future to support regional and local authorities in promoting energy efficiency with their citizens. Examples of such initiatives include the CoM initiative with the potential of reaching out to more than 10 000 cities, thereby providing the possibility to support or even mandate the setup of dedicated financial schemes. The overview of financial instruments developed in the context of the CoM to support energy renovation in buildings shows that local authorities mostly targeted municipal buildings and to a lesser extent private buildings. However, being the level of governance closest to citizens, local authorities may have a significant potential to tap into also in relation to private buildings renovation, as can be seen from the good practices presented above. Financing schemes prove to be particularly effective (e.g. in terms of number of successful applications) when they are accompanied by tailored communication campaigns, aimed at listening to citizens’ preferences and concerns and accompanying them in and simplifying the process. Cooperation among regional and local authorities should also be further encouraged: as seen in previous studies on the CoM, regional authorities may aggregate project initiatives from municipalities to develop investment programmes of the required size to apply for various types of financial support and to become interesting for ESCOs. Finally, regional and local authorities can play a unique role by bringing together a wide range of stakeholders including banks and various professionals in the construction sector. To this end, OSSs can play a crucial role in forging a link between the demand and supply sides of the traditional renovation value chain and supporting building owners in the renovation journey from start to finish. Together with households, local businesses and public authorities, a close stakeholder collaboration facilitated by these bodies can be key to the successful implementation of financing schemes and acceleration of the energy transition of cities and regions.

## Annex

See Table 4.

**Table 4:** Distribution of financial instruments under the CoM initiative (2030) by: (a) type policy and (b) type of building

		(a) Type of policy					(b) Type of building		
		Grants and subsidies	Third party financing	Energy suppliers obligations	Energy and carbon taxes	Public procurement	Residential buildings	Tertiary buildings	Municipal buildings
Austria	Number	2	0	0	0	0	2	0	0
	Share	100%	0%	0%	0%	0%	100%	0%	0%
Belgium	Number	185	18	22	0	145	148	18	204
	Share	50%	5%	6%	0%	39%	40%	5%	55%
Croatia	Number	12	3	0	3	8	6	4	16
	Share	46%	12%	0%	12%	31%	23%	15%	62%
Czechia	Number	2	0	0	0	5	2	0	5
	Share	29%	0%	0%	0%	71%	29%	0%	71%
Finland	Number	0	0	0	0	4	1	0	3
	Share	0%	0%	0%	0%	100%	25%	0%	75%
France	Number	3	1	0	0	4	3	1	4
	Share	38%	13%	0%	0%	50%	38%	13%	50%
Germany	Number	0	0	0	0	3	2	1	0
	Share	0%	0%	0%	0%	100%	67%	33%	0%
Greece	Number	11	2	0	0	9	4	3	15
	Share	50%	9%	0%	0%	41%	18%	14%	68%
Hungary	Number	10	2	0	0	20	9	2	21
	Share	31%	6%	0%	0%	63%	28%	6%	66%
Ireland	Number	13	1	0	0	4	3	7	8
	Share	72%	6%	0%	0%	22%	17%	39%	44%
Italy	Number	89	64	14	0	189	89	36	231
	Share	25%	18%	4%	0%	53%	25%	10%	65%
Latvia	Number	1	0	0	0	3	0	0	4
	Share	25%	0%	0%	0%	75%	0%	0%	100%
Portugal	Number	2	7	0	0	2	3	3	5
	Share	18%	64%	0%	0%	18%	27%	27%	45%
Romania	Number	42	6	0	0	8	7	33	16
	Share	75%	11%	0%	0%	14%	13%	59%	29%
Spain	Number	26	12	24	6	72	37	18	85
	Share	19%	9%	17%	4%	51%	26%	13%	61%
Sweden	Number	0	0	0	0	1	0	0	1
	Share	0%	0%	0%	0%	100%	0%	0%	100%
EU27	Number	398	116	60	9	477	316	126	618
	Share	38%	11%	6%	1%	45%	30%	12%	58%

See Table 5.

**Table 5:** Regional and local schemes that were associated with large energy savings or budgetary resources and were reported to be of high impact in the survey

Region	Covenant involvement as signatory or CTC	ESIF support	Name of scheme	Impact and budget
Vienna region (Austria)	No (City of Vienna is a signatory)	–	Thermal-energetic renovation of residential building (THEWOSAN)	Overall budget: EUR 100 million
Silesia Province (Poland)	No		4.3 Energy efficiency and renewable energy sources in public and residential infrastructure -under the Regional Operational Programme	Overall budget: EUR 303 million Achieved savings: 93,197,833 kWh/year GHG reduction: 37 691,43 t CO2eq/year
Tuscany region (Italy)	No	Yes (ERDF)	Axis 4, Action 4.1.1 Energy efficiency interventions of public buildings Public Energy Announcement	Overall budget: EUR 98 million Achieved savings: 40,000,000 kWh/year GHG reduction: 7.770 t CO2eq/year
Saxony Anhalt (Germany)	No	Yes (ERDF)	Stark III	Overall budget: EUR 121 million GHG reduction: 6.000 t CO2/year
Berlin (Germany)	No (City of Berlin is a signatory)	Yes (ERDF)	Berlin Programme for Sustainable Development (BENE) FS2	Overall budget: EUR 114 million Achieved savings: 100,755,173 kWh/year GHG reduction: 28,853 t CO2/year

## Footnotes

1 Following the withdrawal of the United Kingdom from the Union on the 1 February 2020, the scope of the study was adjusted to cover only the EU27 Member States.

2 www.eumayors.eu

3 https://cohesiondata.ec.europa.eu/themes/4#

4 https://cohesiondata.ec.europa.eu/2014-2020-Categorisation/ESIF-2014-2020-categorisation-ERDF-ESF-CF-planned-/3kkx-ekfq

5 In the period 2014–2020, the CF provided support in Bulgaria, Croatia, Cyprus, the Czech Republic, Estonia, Greece, Hungary, Latvia, Lithuania, Malta, Poland, Portugal, Romania, Slovakia and Slovenia, while the ERDF supported all EU countries. The analysis in the following section does not cover countries that allocated the funds only to **national** programmes.

6 This share can be used for both national and regional programmes.
